# Slot-Coupled Fed 256-Element Planar Microstrip Array with Beam Stability for K-Band Water Level Sensing

**DOI:** 10.3390/s25185904

**Published:** 2025-09-21

**Authors:** Kuang-Hsuan Huang, Yen-Sheng Chen

**Affiliations:** Department of Electronic Engineering, National Taipei University of Technology, 1, Sec. 3, Zhongxiao E. Rd., Taipei 10608, Taiwan

**Keywords:** slot-coupled microstrip arrays, antenna arrays, K-band radar, sidelobe suppression, water-level monitoring

## Abstract

Radar-based water-level monitoring requires antennas with narrow beams, high gain, and low sidelobes. Existing horn and series-fed microstrip arrays either lack compactness or suffer from frequency-dependent beam deviation that reduces sensing accuracy. This paper presents a 256-element slot-coupled planar microstrip array operating in the K-band for water-level radar. The array combines large-scale integration with slot-coupled feeding, which provides inherent 180° phase correction and stabilizes the main beam across frequency. The fabricated array has overall dimensions of 140 mm × 160 mm × 1.12 mm. Simulated results show a peak gain of 22.8 dBi with beamwidths of 5.2° (E-plane) and 4.2° (H-plane), while beam deviation remains within 0.5° across 25.9–27.0 GHz. In comparison, a series-fed array of identical aperture exhibits up to 7.5° deviation and only 15.8 dBi broadside gain. These results demonstrate that the proposed slot-coupled array provides a compact antenna solution meeting regulatory requirements and improving the accuracy of radar-based water-level monitoring systems.

## 1. Introduction

Typhoons and monsoon rains often cause sudden rises in river levels, threatening infrastructure such as bridges and dams. Radar-based water-level monitoring systems have therefore been deployed to provide real-time measurements that support disaster-prevention measures. In these systems, the antenna is critical because its radiation properties determine performance. Narrow beams focus energy on the monitoring zone to improve precision, high gain extends sensing range by strengthening return signals, and low sidelobes suppress interference from multipath and clutter. These requirements are reinforced by regulatory standards; for example, the United States Federal Communications Commission (FCC) specifies K-band operation (24.05–29.00 GHz) with beamwidths under 12° and minimized sidelobe levels (SLLs) [1,2]. Meeting such constraints presents significant challenges for antenna design in practical water-level radars.

Large-scale microstrip arrays are required to satisfy these specifications. A 256-element planar array can form a sub-12° main beam with sufficient gain for long-range K-band sensing, but scaling to this aperture introduces difficulties. Series-fed arrays often exhibit amplitude and phase imbalance, leading to unstable sidelobes and frequency-dependent beam deviation. Planar microstrip- or coaxial-fed arrays improve distribution uniformity but suffer from feeding loss and coupling as size increases. Slot-coupled feeding mitigates these issues by stabilizing current distribution, inherently providing 180° phase correction between adjacent elements, and maintaining broadside radiation across frequency. For water-level sensing, this feature is essential, since frequency-dependent beam drift translates directly into measurement errors. Developing a 256-element slot-coupled planar array with stable frequency response is thus both necessary and challenging.

Commercial designs illustrate this trade-space. Monostatic water-level radars often employ horn antennas for their gain and directivity [3,4,5,6,7,8,9], but horns are bulky and unsuitable for bistatic layouts requiring two apertures. Compact microstrip arrays are therefore preferred [10]. Sidelobe control in such arrays has been pursued using amplitude tapers and window functions. Studies on Kaiser and Blackman weighting have shown reductions for small-to-moderate arrays [11,12,13,14,15,16], and classic windowing theory quantifies their sidelobe behavior [17]. Series-fed arrays use taper optimization methods such as binomial, Dolph–Chebyshev, Taylor, heuristic shaping, and genetic algorithms [18,19,20,21,22,23,24,25]. For example, a 1 × 7 mm-wave array achieved 14.7 dBi gain with –18.3 dB sidelobes using stacked patches and Dolph–Chebyshev weighting [19], while a stacked array at 76–80 GHz achieved sidelobes near –19 dB in both planes [24]. The limitation of series feeding remains beam deviation with frequency. Slot-coupled series-fed architectures address this by relocating the feed to a lower layer and coupling through slots, which impose the required phase relation and suppress beam drift [26,27,28,29,30,31,32,33].

Beyond linear arrays, planar microstrip designs have expanded from 2 × 2 modules. Microstrip-fed versions with optimized power dividers improve sidelobe suppression [34,35]; stacked and slot-coupled radiators widen impedance bandwidth and stabilize radiation [36,37,38,39,40,41]; substrate-integrated waveguide feeding reduces loss and coupling in larger apertures [41]; and substrate-integrated coaxial-line feeding achieves low sidelobes at millimeter-wave frequencies [42]. Genetic algorithms have also been applied to nonuniform amplitude distributions for sidelobe reduction while maintaining gain [43]. Collectively, these works establish options for gain, bandwidth, and sidelobe control across frequency bands relevant to level sensing.

Recent studies further highlight the versatility of slot-coupled structures in high-frequency array design. A waveguide slot-fed patch array has been reported as a compact and efficient radiator suitable for millimeter-wave applications [44]. Work on slot-coupling techniques has extended to stacked-patch antennas integrated in package, demonstrating large-element phased arrays in the W-band [45]. Slot coupling has also been exploited in conformal metasurface-fed arrays to enable efficient beamforming and wide-aperture coverage [46]. In monopulse systems, cavity-backed slot-coupled patch antennas have been shown to achieve high gain, low sidelobes, and polarization control [47]. Investigations into U-slot stacked radiators have demonstrated dual-polarized elements for sub-THz multiple-input multiple-output (MIMO) arrays [48]. In addition, wide-band, wide-beam circularly polarized slot-coupled antennas have been applied to wide-angle scanning arrays, showing the potential for broadband beam agility [49]. Collectively, these works illustrate the breadth of recent international efforts applying slot coupling to array antennas across K-band and higher frequencies, and provide further context for the present work.

Despite these advances, limitations persist. Horn antennas are unsuitable for compact or bistatic systems. Series-fed arrays remain vulnerable to frequency-dependent beam instability. Planar microstrip arrays can deliver wide bandwidths and low sidelobes, but scaling beyond 128 elements is hindered by increased feeding loss and coupling. Few studies address the combined challenge of large-scale integration, slot-coupled feeding, and frequency-stable beam formation in the K-band for water-level radar [50]. Most focus on sidelobe suppression, bandwidth enhancement, or gain, but not on main-beam stability across the operating band. This gap is critical when designing antennas that must comply with FCC regulations while remaining practical in size and manufacturability.

To address these challenges, this paper proposes a 256-element slot-coupled planar microstrip array operating in the K-band. The array achieves narrow beamwidth below 12°, high gain for long-range detection, and stable broadside radiation across 25.6–27.2 GHz. By using slot coupling, the design suppresses frequency-induced beam deviation and reduces side-sector sidelobes (outside ±60°). The antenna is designed and tested, demonstrating its suitability as a compact and regulation-compliant solution for water-level radar.

The remainder of this paper is organized as follows. Section 2 introduces the design theory, including the unit structure, scaling principle, and parametric studies. Section 3 presents the proposed 256-element planar array along with fabrication and results, and provides a performance comparison with a series-fed counterpart. Finally, Section 4 concludes the paper.

## 2. Design Theory

### 2.1. Unit Structure and Radiation Principle

**Figure 1:**
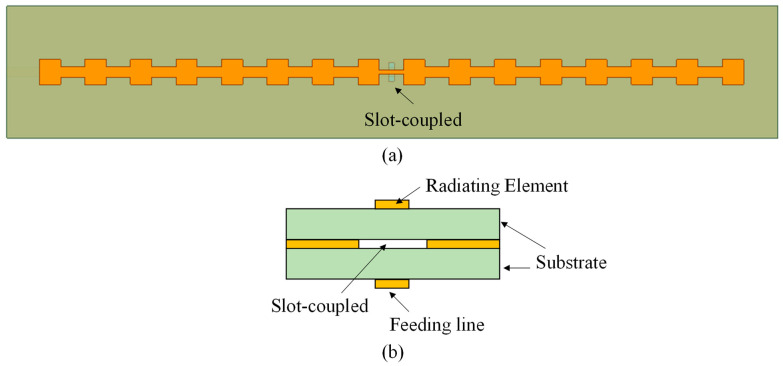
Geometry of the slot-coupled linear patch array: (**a**) top view with slot-coupled arrangement, and (**b**) side view of the three-layer substrate stack.

The slot-coupled patch array operates on a three-conductor stack fabricated on RO4003 laminate (relative permittivity *ε_r_* = 3.38, loss tangent tan*δ* = 0.0027, and thickness 0.508 mm), as shown in Figure 1. The bottom layer carries the microstrip distribution lines, the middle layer is a continuous ground plane containing narrow longitudinal slots, and the top layer consists of rectangular patches forming the radiating chain. The ground plane isolates the distribution network from free space, reducing unintended radiation and coupling between columns. The slot aperture provides the only coupling path between feed and radiator, and its dimensions set the coupling coefficient.

Power on the microstrip excites a strong transverse electric field across the ground-plane slot. In aperture equivalence terms, the slot supports a magnetic surface current that excites the top patches. The two edges of the slot generate equal-magnitude but opposite-polarity fields, producing currents with a 180° phase difference at the feed point. Because excitation occurs locally at each aperture rather than through a long series line, frequency-dependent phase accumulation is minimized, reducing beam squint across 24.05–29.00 GHz.

**Figure 2:**
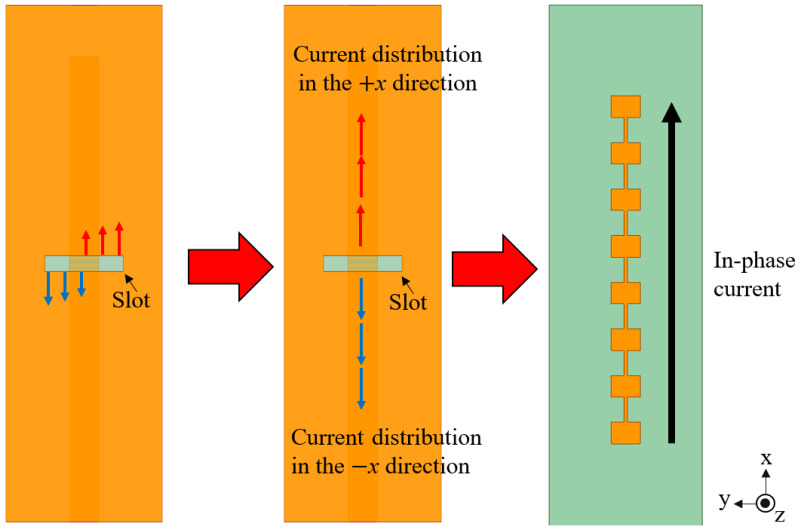
Principle of slot coupling: (**left**) excitation through the slot, (**middle**) opposite currents with 180° shift, and (**right**) resulting in-phase radiation along the array.

Figure 2 illustrates how the slot-induced 180° phase shift interacts with the *π* inversion of the series patch chain. The combination yields in-phase currents toward both +x and −x, supporting a stable broadside beam. A slot-coupled 1-to-2 divider launches equal power into the two branches. Port 1 is the input; Ports 2 and 3 connect to the slot branches feeding the +x and −x directions. The divider achieves |*S*_11_| < −10 dB from 24.16 to 28.34 GHz. At 26.4 GHz, |*S*_21_| = −3.73 dB, |*S*_31_| = −3.80 dB, and the phase difference ∠*S*_21_ − ∠*S*_31_ remains within ±5° over 22.0–31.0 GHz, ensuring balanced two-sided excitation and supporting side-sector SLL suppression.

**Figure 3:**
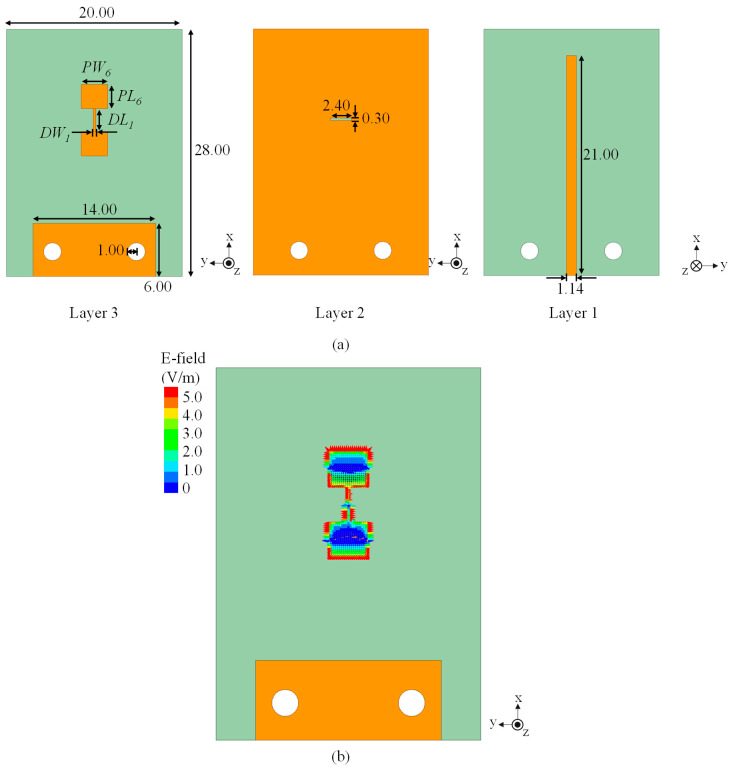
Geometry and field distribution of the two-element slot-coupled array: (**a**) layout with labeled dimensions (unit: mm), and (**b**) simulated electric field distribution. Normalized electric-field distribution is shown on the patch surface. The color bar indicates field magnitude on a linear scale, normalized to the maximum value.

As a starting point, a 1 × 2 slot-coupled fed microstrip antenna was designed to establish the baseline performance. The geometry is shown in Figure 3a. The structure consists of a rectangular slot etched on the middle ground plane, which couples energy from the microstrip feed line on the lower layer to the two radiating patches on the top layer. The patch dimensions are parameterized as *PL*_6_ = 2.75 mm (length) and *PW*_6_ = 3.00 mm (width), while the slot dimensions are *DL*_1_ = 2.60 mm (length) and *DW*_1_ = 0.30 mm (width). To accommodate end-launch connectors, two screw holes are introduced on Layer 1 and Layer 2, and the ground plane is opened with the slot aperture between Layer 2 and Layer 3. Full-wave simulations were performed in HFSS using perfectly matched layer (PML) boundaries in all outward directions to absorb radiated fields. The element spacing is fixed at 0.5*λ* to suppress grating lobes while preserving impedance and coupling balance.

The simulated reflection coefficient of this 1 × 2 structure indicates resonance at 26.4 GHz, where the reflection coefficient reaches −23.5 dB. The −10 dB impedance bandwidth spans 25.90–27.14 GHz, corresponding to a fractional bandwidth of 4.7%. The electric field distribution (see Figure 3b) indicates that both patches radiate in-phase, verifying that the slot provides the required 180° compensation for the natural π inversion of series patches. Strong field concentration around the slot confirms localized coupling, which reduces feedline dispersion effects and stabilizes the broadside beam.

**Figure 4:**
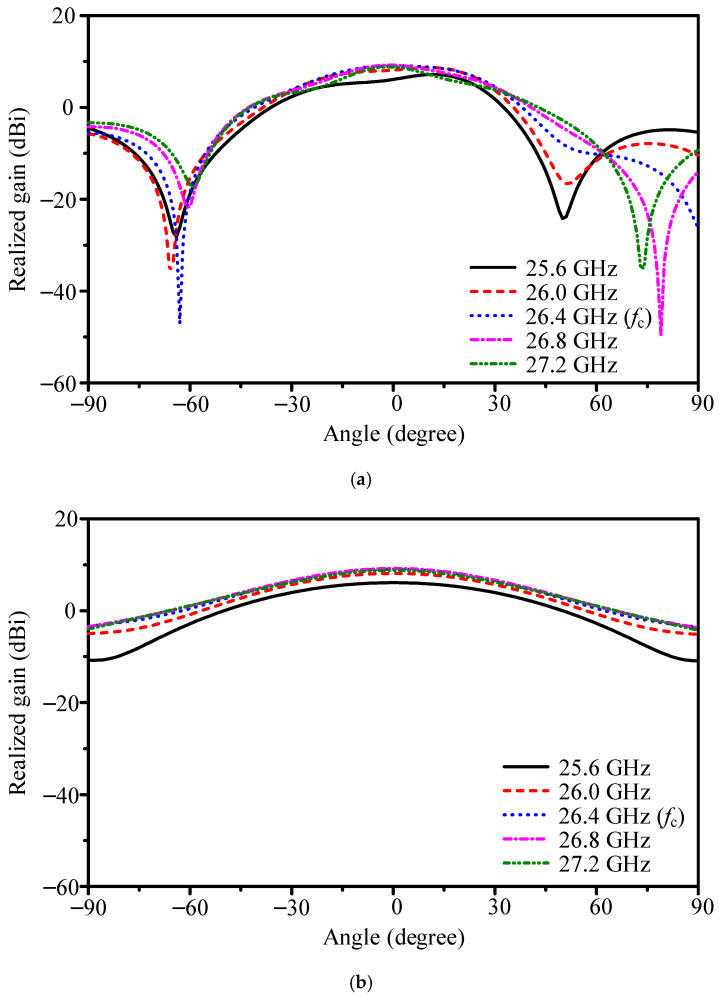
Simulated co-polarized gain patterns of the two-element slot-coupled array across multiple frequencies: (**a**) E-plane and (**b**) H-plane.

Radiation patterns of the 1 × 2 array are shown in Figure 4. With element spacing of 0.48*λ* (5.35 mm) at 26.4 GHz, the antenna achieves 9.0 dBi peak gain and 85.2% efficiency. The half-power beamwidths (HPBWs) are 48.7° (E-plane) and 64.9° (H-plane), with a front-to-back ratio (FBR) of 18.9 dB. Across the operating band, the main beam consistently points to broadside, confirming that slot-coupled excitation mitigates frequency-dependent squint. This establishes the feasibility of extending the scheme to larger arrays for water-level sensing.

### 2.2. Scalable Design Principle

**Figure 5:**
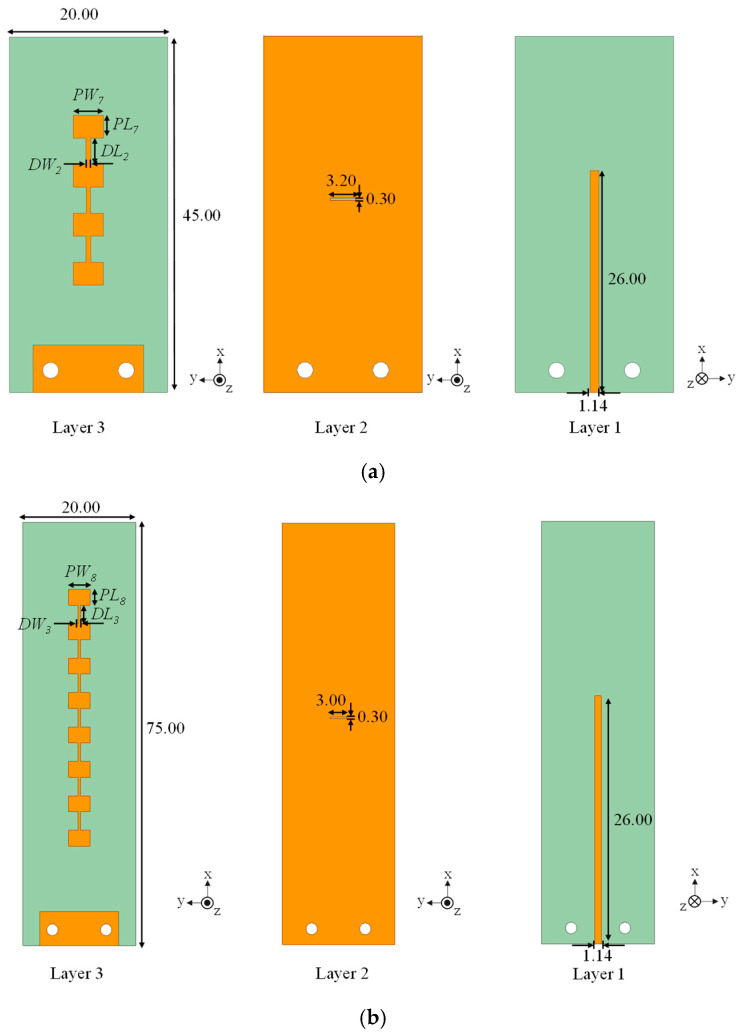

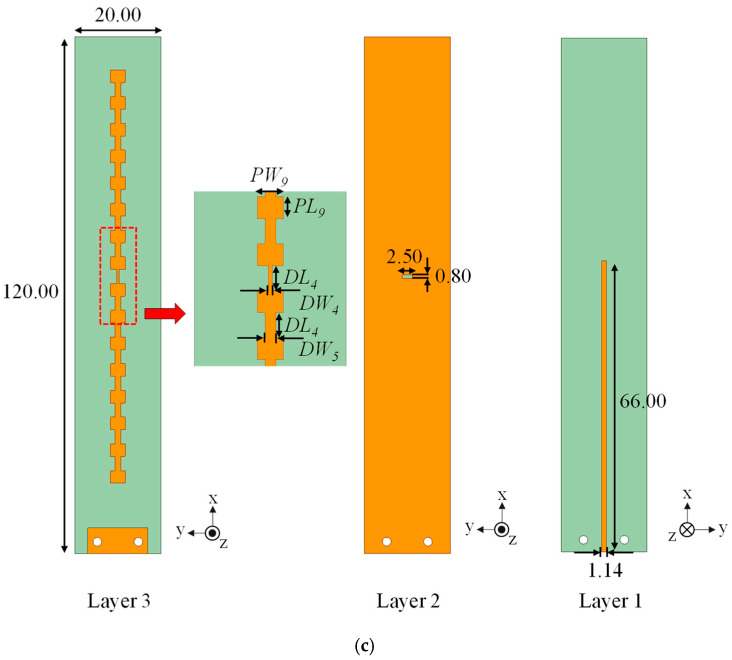
Geometries of the slot-coupled arrays: (**a**) 4-element, (**b**) 8-element, and (**c**) 16-element (unit: mm).

**Figure 6:**
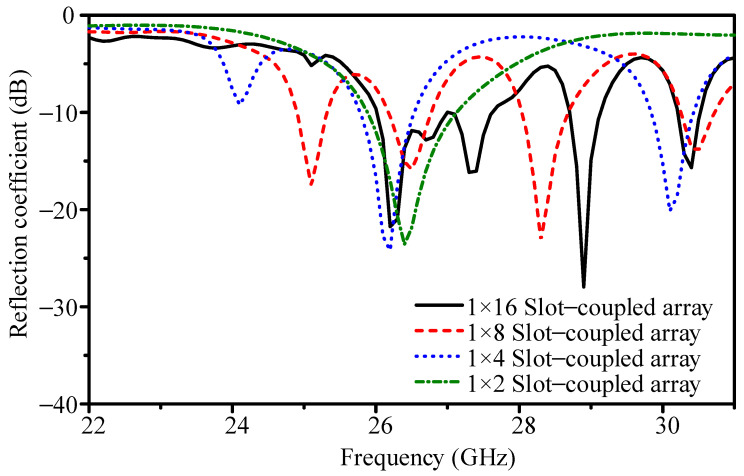
Simulated reflection coefficients of slot-coupled arrays with different element counts.

**Figure 7:**
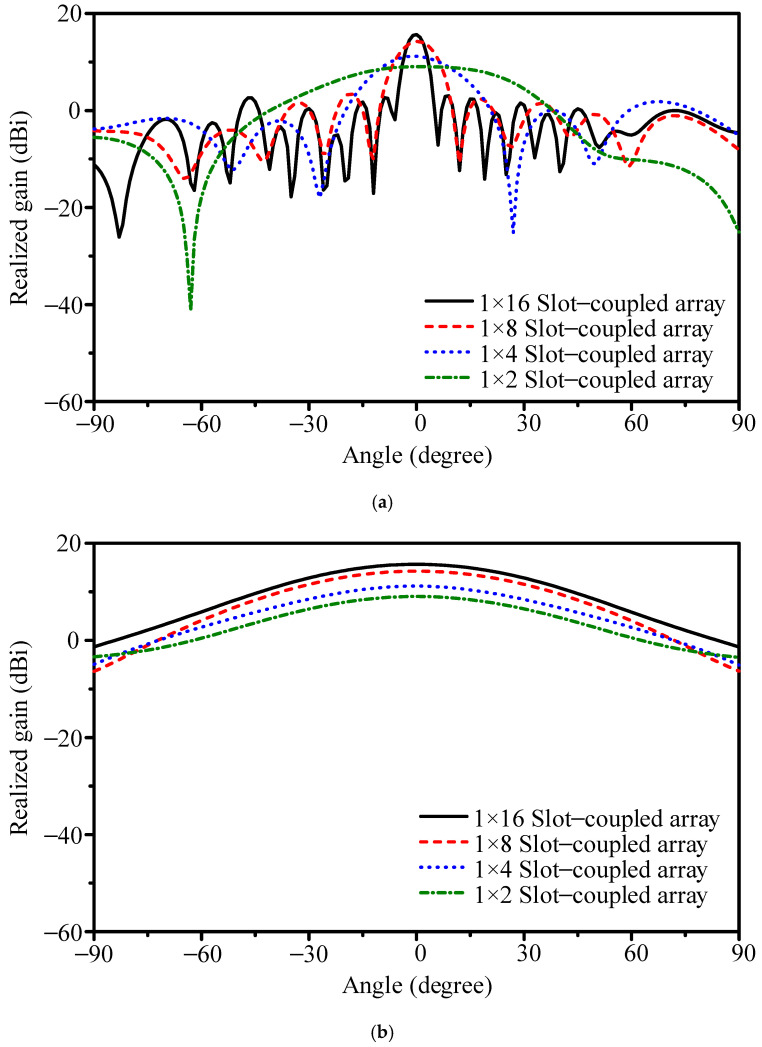
Simulated gain patterns of slot-coupled arrays with different element counts: (**a**) E-plane and (**b**) H-plane.

To reduce the beamwidth of the initial 1 × 2 design, the array was extended to a 1 × 4 slot-coupled series configuration. The geometry is shown in Figure 5a. The patches have dimensions *PL*_7_ = 2.90 mm and *PW*_7_ = 3.80 mm, and the slot measures *DL*_2_ = 3.30 mm in length and *DW*_2_ = 0.60 mm in width. The simulated reflection coefficient in Figure 6 indicates resonance at 26.4 GHz with |*S*_11_| = −13.4 dB. The −10 dB bandwidth is 25.78–26.54 GHz (2.9%). As shown in Figure 7, the 1 × 4 array achieves 11.2 dBi peak gain, 82.6% efficiency, E-plane HPBW of 21.6°, H-plane HPBW of 63.4°, and a FBR of 16.7 dB. Although gain improves, the E-plane HPBW remains wider than the 12° target, motivating further scaling.

The next stage is a 1 × 8 slot-coupled array (Figure 5b). The patch dimensions are *PL*_8_ = 2.80 mm and *PW*_8_ = 3.80 mm, and the slot dimensions are *DL*_3_ = 3.30 mm and *DW*_3_ = 0.50 mm. Figure 6 shows resonance at 26.4 GHz with |*S*_11_| = −15.1 dB and −10 dB bandwidth of 26.17–26.77 GHz (2.3%). With element spacing of 0.54*λ* (6.10 mm), the simulated peak gain is 14.3 dBi and efficiency is 77.9%. The E-plane HPBW narrows to 10.7°, the H-plane HPBW is 63.1°, and the FBR improves to 25.1 dB.

It is noted that the 1 × 8 configuration was chosen as an intermediate step between the 1 × 4 and 1 × 16 arrays. This allowed us to evaluate how doubling the aperture length influences impedance bandwidth, beamwidth, and sidelobe levels, and to verify that the <12° beamwidth requirement could already be achieved at this stage. The results confirmed that the 1 × 8 design provides 10.7° E-plane HPBW with sufficient gain, while motivating further extension to 16 elements to increase robustness against fabrication and frequency variations.

Doubling the length to form a 1 × 16 array introduces broader impedance bandwidth and narrower beams. The geometry is shown in Figure 5c. The patches are *PL*_9_ = 2.90 mm and *PW*_9_ = 3.40 mm, while the slot employs a dual-width structure (*DL*_4_ = 3.30 mm, *DW*_4_ = 0.50 mm, and *DW*_5_ = 1.40 mm). Figure 6 shows that |*S*_11_| reaches −27.9 dB at 26.4 GHz, and the −10 dB bandwidth expands to 26.01–27.63 GHz (6.0%). This demonstrates that extending the series path improves impedance stability and reduces sensitivity to dimensional variation.

Radiation characteristics also scale predictably. With the same element spacing of 0.55*λ* (6.20 mm), the 1 × 16 array achieves 15.7 dBi gain, 62.3% efficiency, E-plane HPBW of 5.3°, H-plane HPBW of 62.0°, and FBR of 19.5 dB. The narrower beamwidth confirms that aperture extension reduces HPBW approximately as expected, though efficiency decreases compared with the 1 × 8 case (77.9%) due to longer current paths and increased dielectric and conductor losses.

Figure 6 and Figure 7 together show the array evolution. As element count increases, gain rises steadily and the E-plane beam narrows, while side-sector SLLs are gradually suppressed. The broadside beam remains stable across 25.6–26.8 GHz, confirming the slot-coupled excitation mitigates frequency drift. Resonance shifts in the S-parameters correspond to the effective electrical length of the radiating chain. For example, the 1 × 8 array length of 45.50 mm matches half-wavelength multiples at 25.1, 26.5, 28.3, and 30.5 GHz, explaining the higher-order resonances that appear as the array scales.

### 2.3. Parametric Study

**Figure 8:**
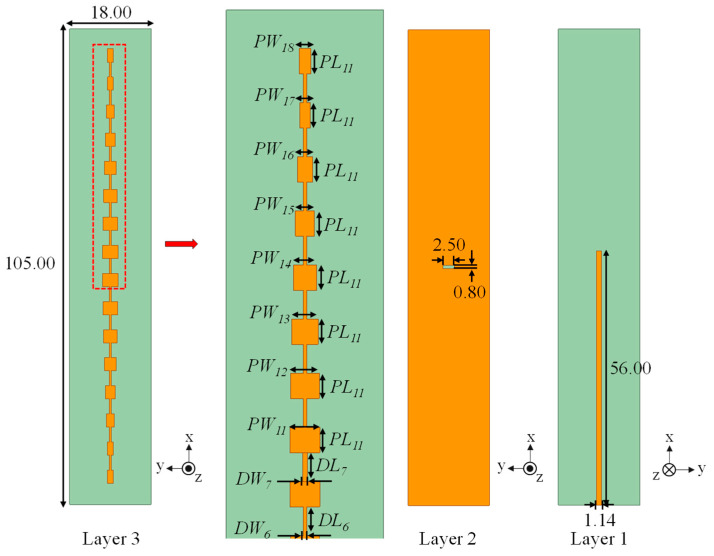
Geometry of the 16-element slot-coupled array used for width and length variation analysis (unit: mm).

After establishing the baseline 1 × 16 slot-coupled array with uniform excitation, alternative amplitude distributions were examined to assess sidelobe suppression. In addition to the uniform case, Dolph–Chebyshev weighting [19,20,21] and Kaiser weighting [12] were implemented. In practice, current tapering can be realized in series-fed microstrip arrays by adjusting patch widths, since radiated current amplitude is proportional to patch width. Figure 8 shows the array layouts for the three distributions.

The normalized amplitude coefficients are listed in Table 1. The uniform distribution applies equal weights, while Dolph–Chebyshev tapers gradually from unity at the center to 0.55 at the edge, and the Kaiser taper decreases more strongly to 0.40 at the edge. The target coefficients were implemented by scaling patch widths element by element.

The detailed geometric parameters of the tapered structures are listed below. For the Dolph–Chebyshev array, the key dimensions were adjusted as follows: *DL*_6_ = 3.300 mm, *DW*_6_ = 0.400 mm, *DL*_7_ = 3.300 mm, *DW*_7_ = 0.500 mm, *PL*_11_ = 2.900 mm, *PW*_11_ = 3.400 mm, *PW*_12_ = 3.264 mm, *PW*_13_ = 2.992 mm, *PW*_14_ = 2.618 mm, *PW*_15_ = 2.176 mm, *PW*_16_ = 1.700 mm, *PW*_17_ = 1.244 mm, and *PW*_18_ = 1.360 mm. For the Kaiser distribution, the optimized set was *DL*_6_ = 3.600 mm, *DW*_6_ = 0.500 mm, *DL*_7_ = 3.300 mm, *DW*_7_ = 0.900 mm, *PL*_11_ = 2.950 mm, *PW*_11_ = 3.400 mm, *PW*_12_ = 3.332 mm, *PW*_13_ = 3.196 mm, *PW*_14_ = 3.026 mm, *PW*_15_ = 2.788 mm, *PW*_16_ = 2.516 mm, *PW*_17_ = 2.210 mm, and *PW*_18_ = 1.870 mm. These values ensured that the designed current ratios matched the target tapers.

**Figure 9:**
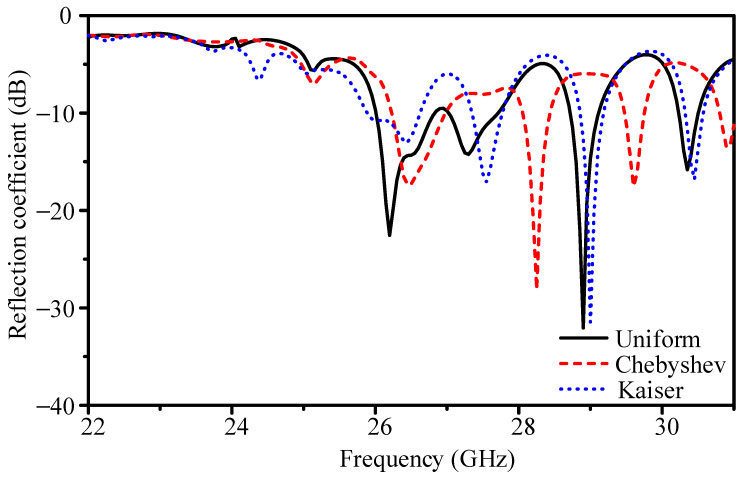
Simulated reflection coefficients of the 16-element slot-coupled array with different current distributions.

Figure 9 shows the reflection coefficients. The uniform array resonates at 26.4 GHz with |*S*_11_| = −14.6 dB and −10 dB bandwidth of 26.01–26.83 GHz (3.1%). The Dolph–Chebyshev taper improves |*S*_11_| to −16.6 dB, but narrows the bandwidth to 26.23–26.98 GHz (2.8%). The Kaiser case yields |*S*_11_| = −12.9 dB and bandwidth of 25.89–26.62 GHz (2.8%). Thus, tapering alters impedance matching only slightly.

**Figure 10:**
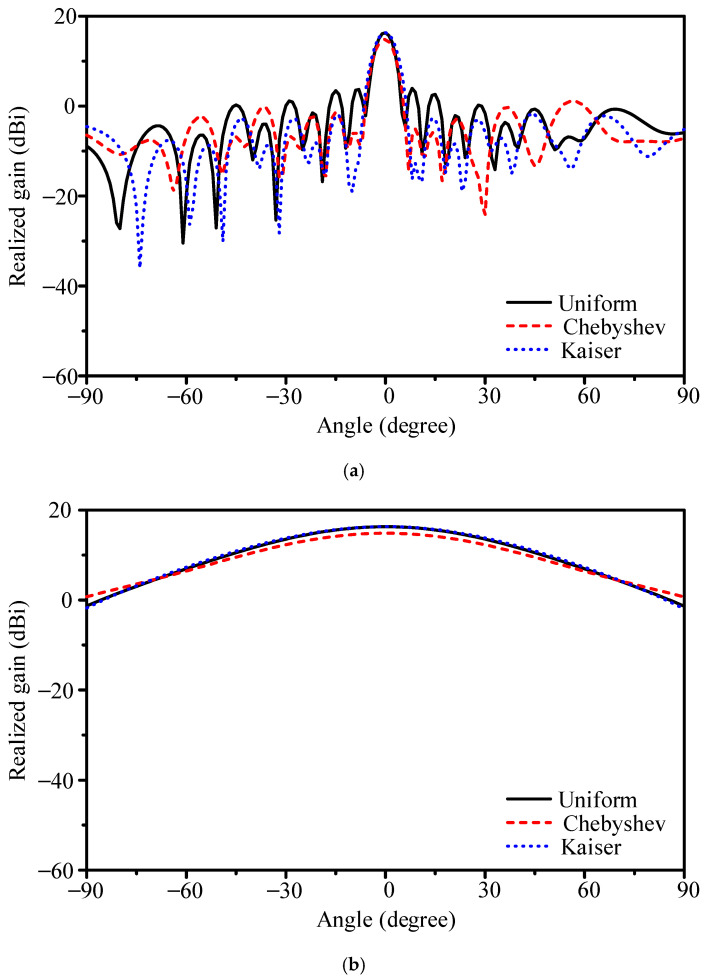
Simulated gain patterns of the 16-element slot-coupled array under different current distributions: (**a**) E-plane and (**b**) H-plane.

The radiation patterns are shown in Figure 10. The uniform case achieves 16.3 dBi gain with 5.1° E-plane HPBW. The Dolph–Chebyshev design reduces gain to 14.9 dBi with HPBW broadened to 5.8°, while the Kaiser design preserves gain at 16.3 dBi but with HPBW widened to 6.1°.

Sidelobe performance shows mixed trends. Beyond ±60°, sidelobe levels do not decrease and may rise by up to 3.0 dB. However, the first sidelobe is clearly reduced: from −12.4 dB (uniform) to −13.7 dB (Dolph–Chebyshev) and −18.4 dB (Kaiser). This indicates that current tapering is effective for suppressing the first sidelobe, though less effective for wide-angle radiation.

**Figure 11:**
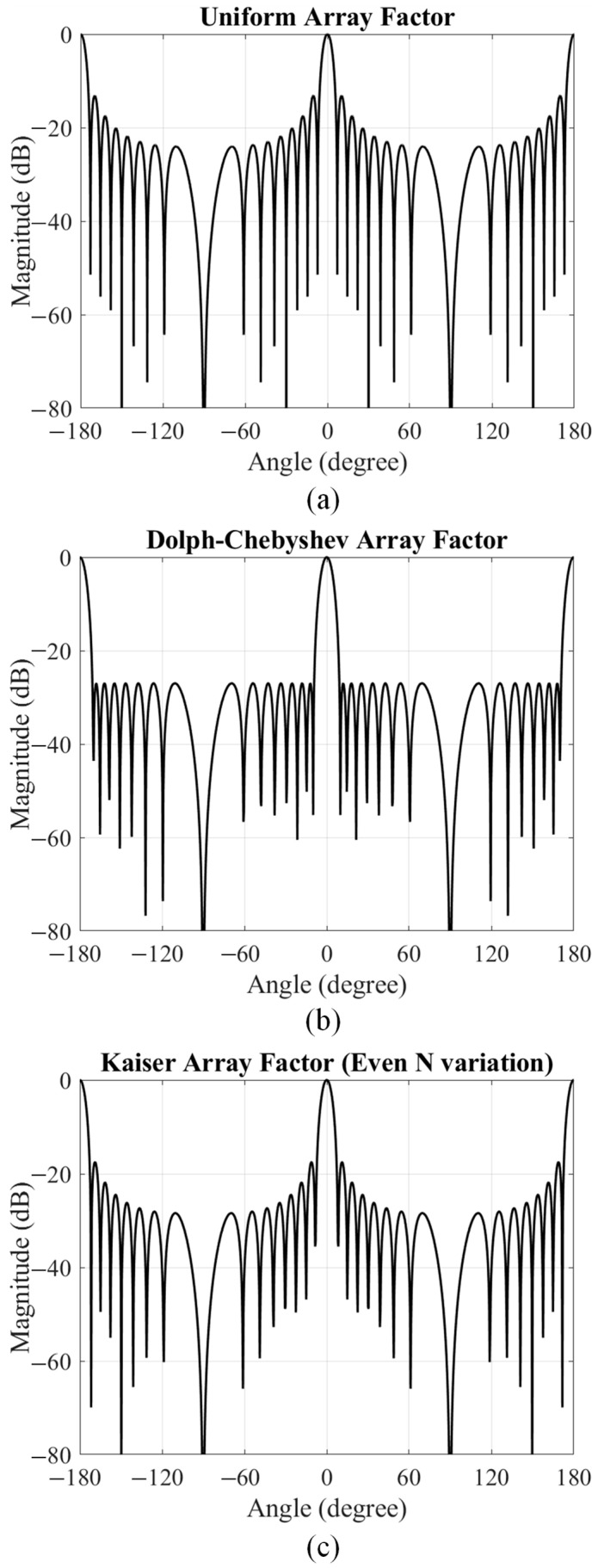
Ideal array-factor patterns for a 16-element linear array under uniform, Dolph–Chebyshev, and Kaiser amplitude distributions.

To support the parametric study, we computed ideal array-factor patterns for a 16-element linear array with *d* = 0.5*λ* under three amplitude distributions: uniform, Dolph–Chebyshev (equal-ripple sidelobes set to −27 dB), and Kaiser taper (even number realization). The results are shown in Figure 11.

For the uniform distribution, the HPBW is about 6.0°, but the first sidelobe is relatively high at −13.2 dB around ±10.5°, with the second sidelobe at −17.5 dB near ±18.1°. With Dolph–Chebyshev weighting, the HPBW broadens to 8.0°, while both the first and second sidelobes are uniformly suppressed near −27.0 dB (at ±12.0° and ±18.2°, respectively). The Kaiser taper gives an HPBW close to 6.0° and reduces the first sidelobe to −17.6 dB near ±11.0°, with the second sidelobe at −21.9 dB near ±18.6°.

These array-factor patterns clearly demonstrate the trade-off: tapering suppresses sidelobes at the expense of a wider main beam, while uniform excitation maintains the narrowest main lobe but with higher sidelobes. Differences between Figure 11 and the full-wave simulated results arise from element radiation patterns, mutual coupling, and the practical realization of amplitude tapers by adjusting patch widths.

In summary, non-uniform excitations reduce the first SLL but at the expense of bandwidth or gain. For water-level radar, where both narrow beamwidth and high efficiency are required, the uniform distribution remains the most suitable choice despite its higher sidelobe level.

## 3. Proposed Antenna and Performance

### 3.1. 256-Element Planar Array

**Figure 12:**
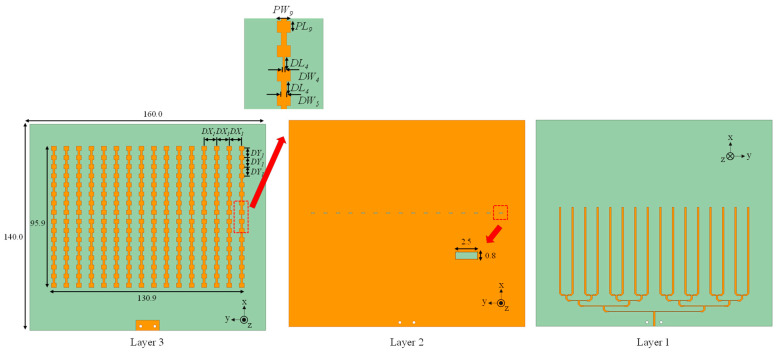
Geometry of the proposed 256-element slot-coupled planar array (unit: mm).

Building on the uniform 1 × 16 slot-coupled linear array from Section 2, the proposed antenna forms a 16 × 16 planar aperture by tiling sixteen identical columns. Each column retains the slot-coupled series-patch topology, preserving in-phase current excitation and stable broadside radiation. Figure 12 shows the overall layout and layer views. The column pitch and inter-element spacing are inherited from the linear design to maintain a narrow E-plane beam, suppress radiation outside ±60°, and avoid grating lobes across the band.

The overall dimensions are 140.00 mm × 160.00 mm × 1.121 mm, which corresponds to 12.33*λ* × 14.10*λ* × 0.10*λ* at 26.4 GHz. All radiators use the same unit cell as the uniform 1 × 16 design. The top-layer patch dimensions are *PL*_9_ = 2.90 mm and *PW*_9_ = 3.40 mm. The ground plane contains a longitudinal slot with a dual-width transition (*DL*_4_ = 3.30 mm, *DW*_4_ = 0.50 mm, and *DW*_5_ = 1.40 mm). The array is fabricated on RO4003 laminate using a three-metal stack: bottom layer for microstrip distribution, middle ground with slots, and top layer with series patch chains. The inter-element spacing matches the validated 1 × 16 case so that the synthesized beamwidth meets the <12° requirement and the main beam remains at broadside across the operating band.

**Figure 13:**
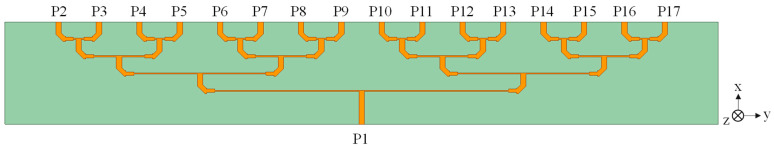
Microstrip T-junction feeding network for the 256-element planar array.

A hierarchical T-junction microstrip tree distributes power equally to the sixteen columns (Figure 13). The input port is located at the lower edge of the board. Equal-length branches divide power into left and right halves, followed by second-level tees until each column is fed. Junction widths and line impedances are designed for matching, ensuring equal amplitude and phase at all outputs. Ports are defined as follows: Port 1 is the array input at the tree root; Ports 2–17 are the column feeds; each column then uses its slot feed to excite the 16-element series chain.

### 3.2. Fabrication and Results

**Figure 14:**
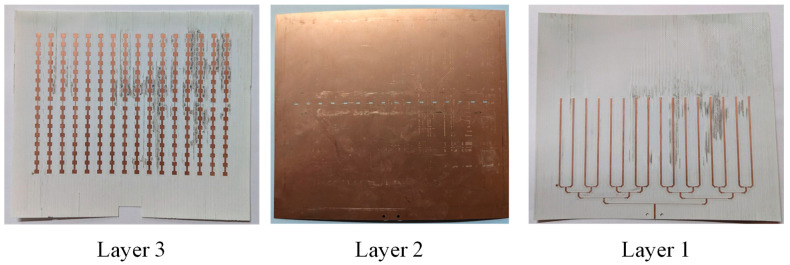
Fabricated 256-element slot-coupled planar array prototype.

The 16 × 16 slot-coupled planar array was fabricated and tested. Figure 14 shows the prototype: the left panel presents the 256 top-layer patches, the middle panel shows the ground plane with etched slots, and the right panel illustrates the microstrip T-junction network on the bottom side. The bottom board contains Layer 1 (feeding network) and Layer 2 (ground with slots), while the top board carries Layer 3 (patch radiators). The two boards were aligned and bonded with a 0.05 mm double-sided adhesive film, and the edges were reinforced with tape to prevent deformation during measurement.

**Figure 15:**
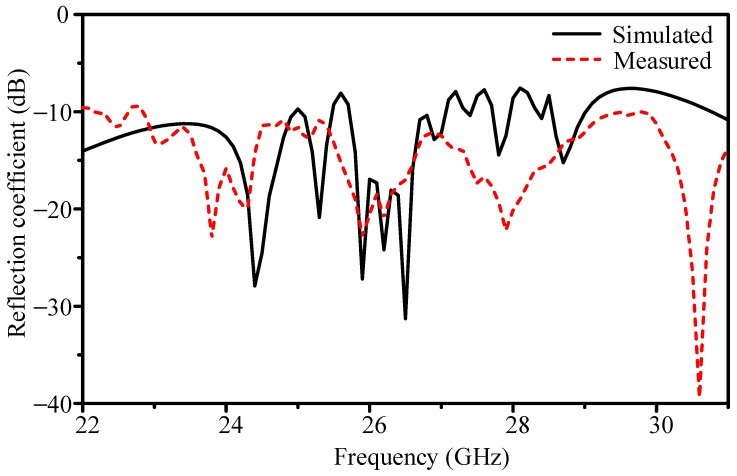
Simulated and measured reflection coefficients of the proposed 256-element slot-coupled planar array.

The measured reflection coefficient is compared with simulation in Figure 15. The simulation shows |*S*_11_| = −18.6 dB at 26.4 GHz, with −10 dB bandwidth of 25.72–27.06 GHz (5.1%). The measurement shows a similar resonance, with |*S*_11_| = −17.5 dB minimum, but a broader −10 dB bandwidth of 22.90–29.70 GHz (26.1%).

The simulated and measured reflection coefficients in Figure 15 show the same resonance near 26.4 GHz and very similar overall trends. Using the −10 dB return-loss threshold, the simulated fractional bandwidth is 25.72–27.06 GHz (5.1%), whereas the measured response spans 22.90–29.70 GHz (26.1%). This difference is primarily a threshold artifact: in simulation, a few edge frequencies rise slightly above −10 dB, which compresses the reported bandwidth; the measured curve stays below −10 dB at those edges, so the span appears much wider. If the matching threshold is relaxed to VSWR 2.5:1 (−7.4 dB), the simulated and measured fractional bandwidths closely agree. Likewise, considering multiple simulated frequency segments where |*S*_11_| is already below −10 dB (instead of only the single contiguous region around 26.4 GHz) yields an effective simulated coverage substantially larger than 5.1%. Operationally, the measured 3 dB gain bandwidth of 25.9–26.6 GHz confirms the intended operating window for the radar system. Additional broadening in measurement can be attributed to practical effects—minor interlayer alignment tolerances, adhesive-film thickness variation, and connector/launch parasitics—which were not fully included in the simulation model.

**Figure 16:**
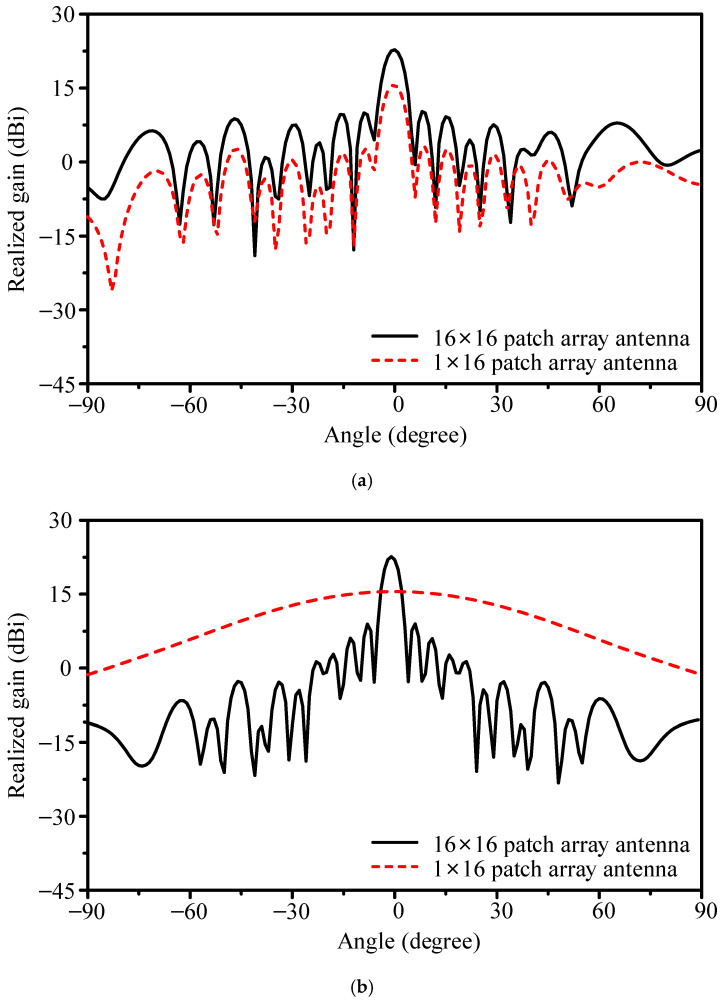
Simulated gain patterns of the 256-element planar array compared with the 16 × 1 linear array: (**a**) E-plane and (**b**) H-plane.

Figure 16 presents the simulated radiation patterns. At 26.4 GHz, the array achieved 22.8 dBi gain, with HPBWs of 5.2° in the E-plane and 4.2° in the H-plane. The FBR was 24.1 dB, and sidelobe levels beyond ±60° remained below −18.7 dB. For comparison, the 16 × 1 array provided 15.6 dBi gain. Thus, the planar 16 × 16 design improves gain by 7.2 dB while maintaining narrow beams in both planes. Across 24.1–29.0 GHz, efficiency ranged from 53.0% to 68.0%. The maximum gain of 23.57 dBi occurred at 26.2 GHz, with 3 dB gain bandwidth of 25.9–26.6 GHz.

**Figure 17:**
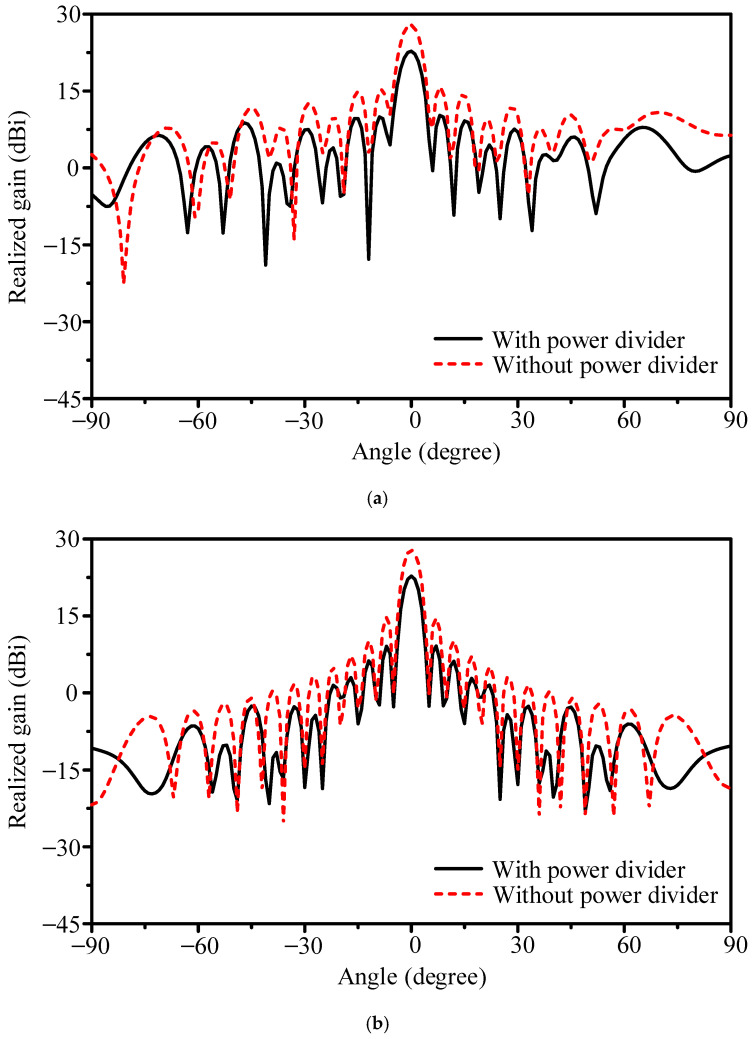
Simulated gain patterns of the planar array with different feeding schemes: (**a**) E-plane and (**b**) H-plane.

Theoretically, scaling from 16 × 1 to 16 × 16 should provide about 12 dB gain increase. Only 7.2 dB was observed, indicating ~5 dB loss in the feeding structure. To verify this, two cases were compared: (1) the fabricated array using the T-junction divider and (2) an idealized array with all 16 column ports excited directly by equal-amplitude signals. As shown in Figure 17, the proposed design achieved 22.8 dBi at 26.4 GHz, while the idealized case reached 27.9 dBi. The 5.1 dB discrepancy is attributed to loss and mismatch in the 1-to-16 microstrip divider.

**Figure 18:**
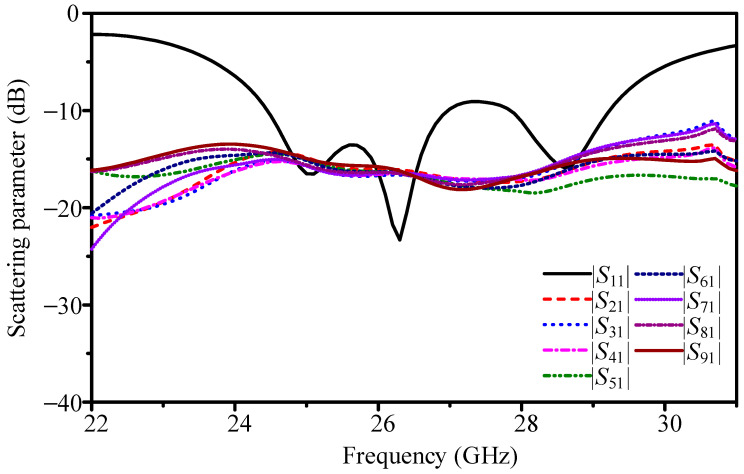
Simulated per-port transmission of the full 1-to-16 divider.

To quantify the loss introduced by the feeding network, we first analyze the elementary 1-to-2 divider. Its simulated S-parameters show |*S*_11_| < −10 dB from 22.0 to 31.0 GHz. At 26.4 GHz, the two paths exhibit |*S*_21_| ≈ −4.3 dB and |*S*_31_| ≈ −4.3 dB, with output-to-output mutual coupling |*S*_32_| ≈ −6.4 dB. Relative to the −3.0 dB ideal split, this corresponds to about 1.3 dB excess insertion per stage. Cascading four such stages yields an expected penalty near 5.2 dB for a 1-to-16 divider. Direct simulation of the complete 1-to-16 network, as shown in Figure 18, gives per-port |*S*_21_| between −16.1 and −16.7 dB at 26.4 GHz; since the ideal equal split is −12.0 dB, the additional loss is 4.1–4.7 dB (typ. 4.4 dB). The network itself is well matched (|*S*_11_| below −20 dB at 26.4 GHz), indicating that the dominant deficit is dissipative and junction-related rather than reflective. This network-level result is consistent with the array-level comparison in Figure 17, where the prototype with the T-junction tree yields 22.8 dBi, while the ideal equal-excitation case reaches 27.9 dBi, i.e., 5.1 dB difference.

To reduce this penalty, several practical strategies can be considered in future work. One option is to implement the main distribution network on a lower-loss or hybrid medium, such as stripline, substrate-integrated waveguide (SIW), or substrate-integrated coaxial line (SICL), while retaining short microstrip transitions only near the radiating slots. This modification directly targets the dielectric and conductor attenuation that accumulates along the long trunks of the four-stage divider. Another improvement is to replace the simple T-junction dividers with isolation-matched splitters such as Wilkinson or multi-section designs incorporating thin-film resistors. These alternatives provide better output isolation, suppress passband ripple, and reduce junction-related insertion loss, all while preserving the −12 dB equal-power distribution to each port. Additional geometric refinements can further reduce loss. Relocating the feed point toward the center of the array or adopting dual-edge feeding decreases the average trunk length, while the use of mitered bends, via fences, and wider metal traces with thicker copper lowers conductor losses in the high-current regions. Finally, optimization of the connector and launch transition can suppress residual mismatch and ripple caused by the pad and via layout.

### 3.3. Performance Comparison

**Figure 19:**
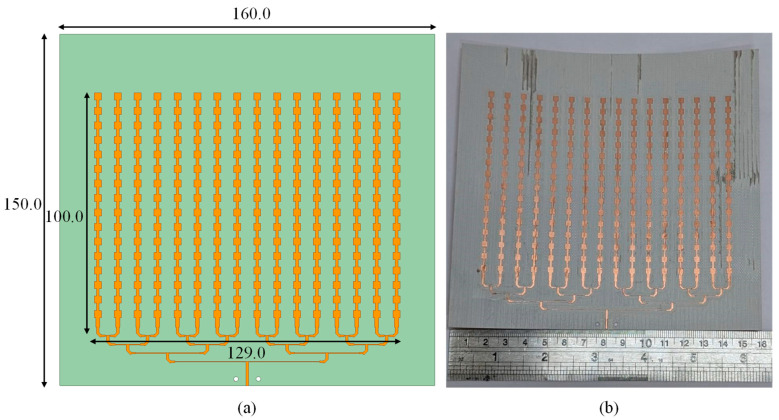
Geometry of the series-fed patch array: (**a**) layout with dimensions (unit: mm), and (**b**) fabricated prototype [50].

For comparison, a 16 × 16 series-fed microstrip array was designed and fabricated under the same aperture size and using the same RO4003 substrate [50]. The only difference lies in the feeding scheme. Unlike the slot-coupled array, the series-fed version employs a single dielectric layer, producing a thinner profile but without the phase compensation mechanism provided by the slot. Figure 19 shows the geometry and fabricated prototype. Each branch is implemented as a continuous series chain of 16 uniformly sized patches, and all 16 branches are combined at the lower edge of the aperture.

At 26.4 GHz, simulation shows peak gain of 24.6 dBi, efficiency of 68.8%, E-plane HPBW of 5.6°, H-plane HPBW of 4.2°, and FBR of 31.7 dB. However, measurement shows a large discrepancy: at 26.5 GHz, the maximum gain is 15.8 dBi in the E-plane (at +1°) and 16.1 dBi in the H-plane (at −1°), both with HPBW ≈ 5.0° [50].

**Figure 20:**
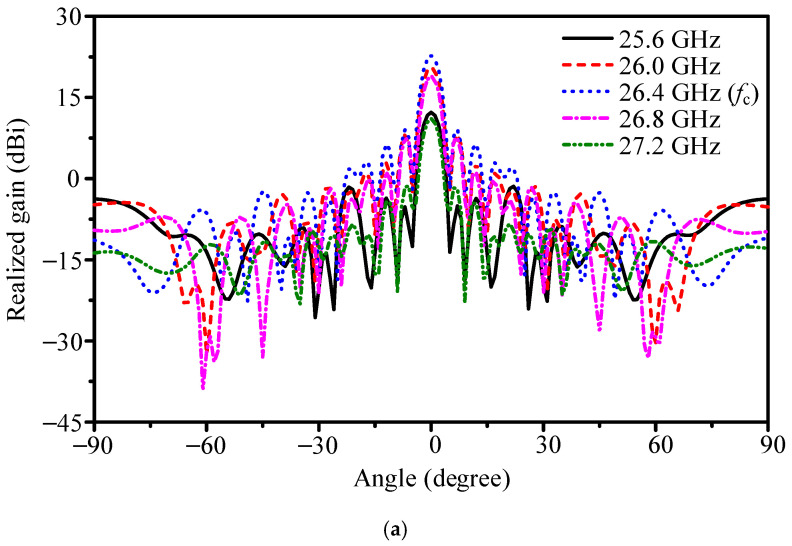

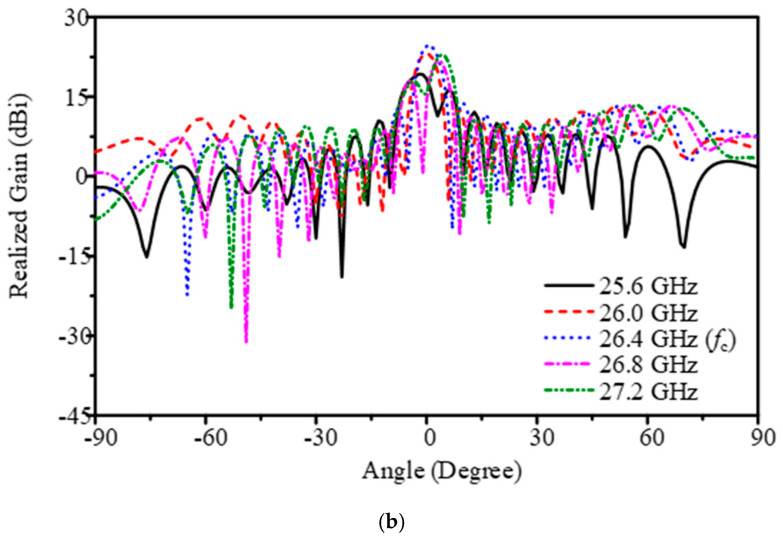
Gain patterns across frequencies: (**a**) slot-coupled 256-element array and (**b**) series-fed counterpart.

The difference between the two architectures is shown in Figure 20. For the slot-coupled array, the main beam remains at broadside across 25.9–27.0 GHz, with variation under 0.5°. In contrast, the series-fed array exhibits progressive beam tilt with frequency; at 27.2 GHz, the deviation reaches 7.5°. Such angular drift severely affects radar sensing: effective gain in the desired direction decreases, and the system becomes vulnerable to range bias and reduced detection probability. Although the simulated broadside gain of the series-fed design is higher, the broadside response collapses off the design frequency due to beam squint, yielding lower usable gain in practice.

The comparison highlights a fundamental trade-off. The slot-coupled array provides stable broadside radiation across the required K-band window, achieving 22.8 dBi gain and side-sector sidelobes below −18.6 dB, but at the cost of a thicker profile and loss in the 1-to-16 divider. The series-fed array, though thinner and theoretically capable of 24.6 dBi gain, suffers from strong frequency-dependent beam deviation that undermines sensing reliability. For water-level monitoring, where consistent pointing to the water surface is essential, the slot-coupled architecture offers the more practical and reliable solution.

From a system perspective, the antenna characteristics translate directly into improved water-level sensing accuracy. The narrow main beams reduce the illuminated footprint on the water surface, allowing finer range resolution. High gain strengthens the radar echo, which is essential for reliable detection over long river spans and reservoirs. The low sidelobe levels minimize returns from riverbanks, bridges, and other clutter sources, thereby enhancing measurement robustness. Most importantly, the frequency-stable main beam ensures that level readings are not biased by angular drift, a problem observed in conventional series-fed arrays. These factors collectively confirm that the proposed array fulfills the operational needs of radar-based water-level monitoring.

The proposed array can also be compared with representative antenna solutions reported in the literature. Compared with horn antennas [3,4,5,6,7,8,9], which are commonly adopted in commercial level radars, the proposed planar array achieves a similar order of beam sharpness and gain while avoiding the bulk and depth of horn profiles. This compactness is not only advantageous for installations on bridges or dam surfaces but is essential for bistatic layouts, where two apertures must be accommodated within limited space. Relative to series-fed microstrip arrays [18,19,20,21,22,23,24,25,50], the improvement is more fundamental. Although such arrays can provide narrow beams and even employ tapering to lower sidelobes, their reliance on cumulative phase along the feedline makes the main beam highly sensitive to frequency. Reports of squint up to several degrees [22,25,50] illustrate this limitation. In water-level sensing, even a few degrees of drift can directly bias the inferred water height. By contrast, the proposed slot-coupled excitation provides inherent 180° phase correction, and the main beam remained within 0.5° of broadside across 25.9–27.0 GHz. Other planar microstrip approaches [34,35,36,37,38,39,40,41,42,50] have targeted bandwidth broadening or sidelobe suppression, for example, using stacked patches [36,37,38] or substrate-integrated waveguide feeds [41]. While these methods succeed for small-to-moderate apertures (typically 2 × 2 to 8 × 8), scaling to hundreds of elements introduces feeding loss and coupling that degrade performance [50]. The present work therefore addresses the open challenge of combining large-scale integration with beam stability.

## 4. Conclusions

A 256-element slot-coupled planar microstrip array for K-band water-level radar has been presented. The design combines large-scale integration with slot-coupled feeding to achieve frequency-stable broadside radiation, a capability not attainable with conventional series-fed or planar microstrip arrays. The proposed array maintained its main beam within 0.5° of broadside across 25.9–27.0 GHz, while the series-fed counterpart deviated by up to 7.5°. The simulated patterns show narrow beamwidth below 12°, side-sector sidelobe levels below −18.6 dB, and stable frequency response. In contrast, conventional approaches sacrifice one or more of these requirements. By addressing the trade-off between array scale and beam stability, the slot-coupled design provides a compact antenna solution suitable for precise and regulation-compliant radar-based water-level monitoring.

## Figures and Tables

**Table 1:** Normalized current weighting for 1 × 16 array designs.

Element Index (from Center)	*N* = 8	*N* = 7	*N* = 6	*N* = 5	*N* = 4	*N* = 3	*N* = 2	*N* = 1
Uniform	1.00	1.00	1.00	1.00	1.00	1.00	1.00	1.00
Dolph–Chebyshev	1.00	0.98	0.94	0.89	0.82	0.74	0.65	0.55
Kaiser	1.00	0.96	0.88	0.77	0.64	0.50	0.37	0.40

## Data Availability

The original contributions presented in this study are included in the article. Further inquiries can be directed to the corresponding author.
